# Intestinal tuberculosis with perforation in an immunocompetent adult presenting with chronic abdominal pain: A case report

**DOI:** 10.3892/mi.2025.272

**Published:** 2025-10-01

**Authors:** Hugo Alberto Roblero López, Luisa Fernanda Montemayor Burrola

**Affiliations:** Department of Internal Medicine, General Regional Hospital No. 1, Mexican Institute of Social Security (IMSS), Chihuahua, Chihuahua 31203, Mexico

**Keywords:** intestinal tuberculosis, bowel perforation, extrapulmonary tuberculosis, abdominal pain, immunocompetent host

## Abstract

Intestinal tuberculosis is a rare extrapulmonary manifestation of *Mycobacterium tuberculosis*, often presenting with non-specific symptoms that delay diagnosis, even in immunocompetent individuals. This case report describes the clinical course, diagnostic workup and surgical findings of an immunocompetent adult male who developed intestinal tuberculosis with perforation. The present study reports the case of a 39-year-old male patient with a history of smoking, alcohol consumption, occasional cannabis consumption, poor hygienic practices and a family history of tuberculosis, who developed progressive chronic abdominal pain and significant weight loss. An initial evaluation revealed signs of acute abdomen and cavitary pulmonary lesions. An exploratory laparotomy uncovered an intestinal perforation with caseating granulomas. Histopathological analysis confirmed tuberculosis enteritis with perforation, and anti-tuberculosis therapy was treated with a standard anti-tuberculosis regimen including isoniazid, rifampin, pyrazinamide and ethambutol. The case presented herein underscores the need for the consideration of abdominal tuberculosis in patients with chronic gastrointestinal symptoms, particularly in endemic regions and among individuals with atypical risk factors. It also highlights the diagnostic challenges posed by delayed recognition, exacerbated by the COVID-19 pandemic, and the critical need to strengthen epidemiological surveillance and adherence to prophylactic treatment in exposed individuals. A multidisciplinary approach integrating clinical, radiological and histopathological tools proved essential for accurate diagnosis and comprehensive management.

## Introduction

Tuberculosis is a systemic infection caused by *Mycobacterium tuberculosis*, primarily affecting the pulmonary parenchyma (1). Recognized as a global public health issue (2), it ranks among the top 10 causes of mortality from infectious diseases and is characterized by high transmissibility, potential for dissemination, and significant lethality (3,4). An estimated one-quarter of the world's population is at risk of developing tuberculosis, with ~10 million active cases and 1.4 million related deaths annually attributable to the disease (5). In Mexico, the incidence is 24 cases per 100,000 inhabitants, with up to 80% of strains reported as resistant to standard treatment and ~10% classified as multidrug-resistant (6). Several predisposing factors, such as a low socioeconomic status, overcrowding, substance abuse, homelessness and poor living conditions are associated with an increased risk of the development of the disease (7). Although addressing these social determinants has helped reduce incidence rates, significant challenges remain, particularly related to the delayed diagnosis of new cases, particularly those with atypical clinical presentations (8).

Although pulmonary tuberculosis is the most common clinical manifestation, the hematogenous dissemination of *Mycobacterium tuberculosis* can lead to extrapulmonary forms, with abdominal tuberculosis being the most frequent presentation (9). This extrapulmonary form poses a diagnostic challenge, as it may occur in otherwise healthy individuals presenting with non-specific symptoms, such as fever, diarrhea, constipation and weight loss, often mimicking abdominal or peritoneal malignancies (10,11). Given that misdiagnosis can worsen the clinical course, accurate identification relies on radiological, histopathological and molecular criteria. For these reasons, abdominal tuberculosis is often considered a diagnosis of exclusion (12-14).

However, despite thorough diagnostic efforts, the majority of cases of abdominal tuberculosis without pulmonary involvement often remain undetected, allowing disease progression. In this context, up to 25% of patients develop gastrointestinal tuberculosis complicated by stenosis, and ~20% ultimately require surgical intervention (15). Among the most severe, yet rare complications is intestinal perforation, which can be life-threatening and requires urgent surgical management. Due to its non-specific symptoms and potential severity, abdominal tuberculosis often remains unrecognized. The present case report describes an uncommon manifestation of intestinal tuberculosis in an immunocompetent adult, complicated by bowel perforation. By highlighting the clinical course, diagnostic process and surgical findings, the present study aimed to raise awareness of this rare, yet severe presentation, and emphasize the need to consider abdominal tuberculosis in the differential diagnosis of chronic abdominal pain, particularly in endemic regions.

## Case report

A 39-year-old male patient with a history of chronic tobacco use (25 pack-year index) and habitual alcohol consumption for 23 years, both discontinued 2 years prior to presentation in January, 2025 at Hospital General Regional No 1, Unidad Morelos del Instituto Mexicano Del Seguro Social, Chihuahua, Mexico. He reported occasional cannabis use and multiple tattoos performed under poor hygienic conditions. His family history was notable for the death of a sister due to pulmonary tuberculosis in 2006.

The patient was admitted to the hospital due to the exacerbation of chronic abdominal pain that had persisted for 2 years. The pain was colicky in nature and mild in intensity (3/10 on the visual analog scale), worsened by food intake and improved with fasting. Upon admission, the patient was febrile (38.5˚C), with a heart rate of 112 bpm, blood pressure of 100/65 mmHg, a respiratory rate of 24 breaths per minute and ab oxygen saturation of 94% on room air. He appeared pale, diaphoretic and in visible discomfort. An abdominal examination confirmed moderate distension, involuntary guarding and a board-like abdomen.

Laboratory tests revealed leukocytosis (15,600/µl) with left shift (90% neutrophils), normocytic normochromic anemia (Hb 9.2 g/dl), thrombocytosis (420,000/µl), an elevated erythrocyte sedimentation rate (86 mm/h), a high level of C-reactive protein (132 mg/l) and hypoalbuminemia (2.8 g/dl). Liver and renal function tests yielded results which were within normal limits. HIV testing was negative.

The condition was accompanied by general symptoms, including asthenia, adynamia and decreased appetite. Over the past 3 months, he reported a progressive increase in pain intensity, along with an estimated weight loss of ~20% of his usual body weight. He had previously received symptomatic treatment without significant clinical improvement.

**Figure 1:**
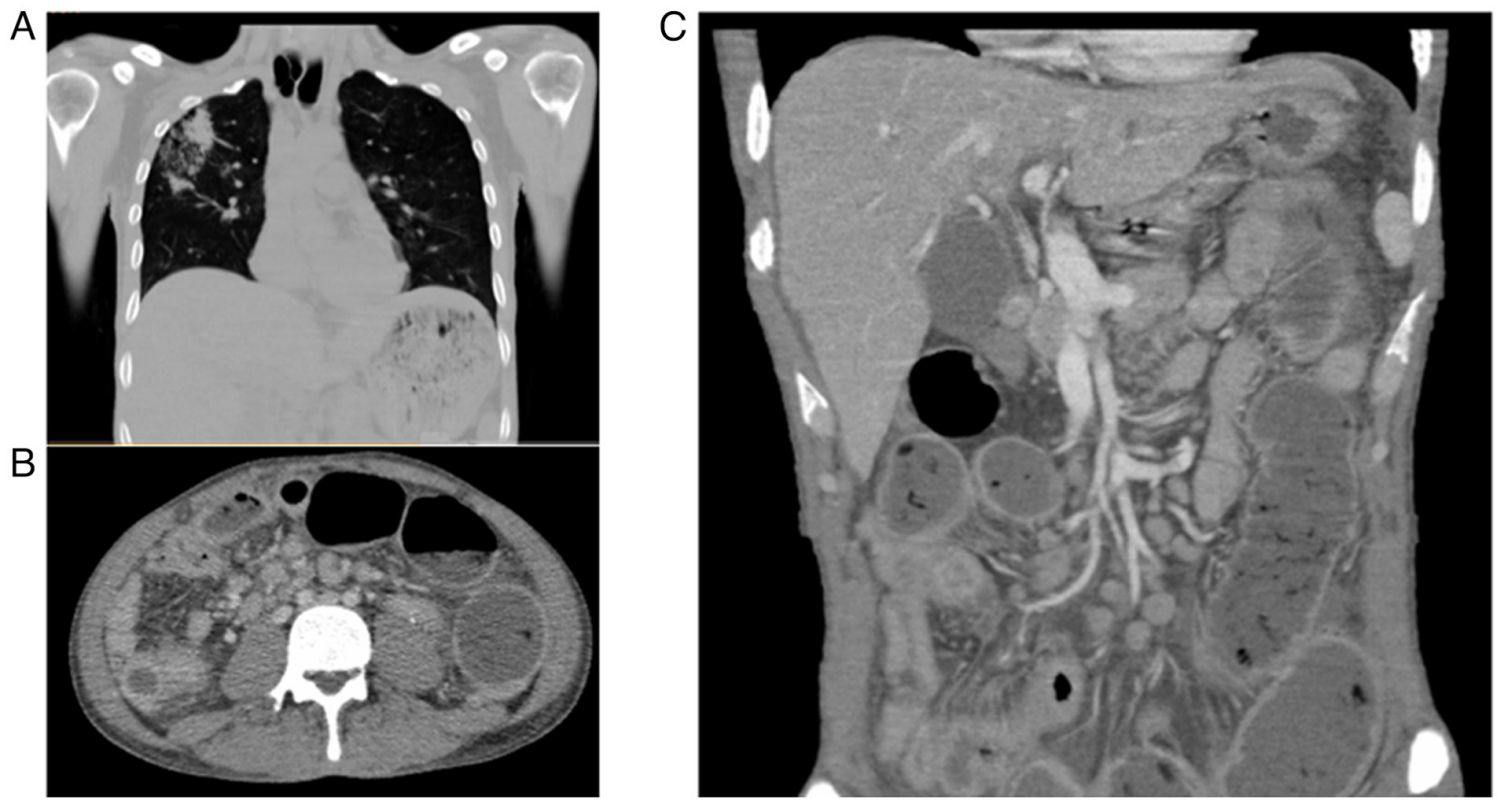
Non-contrast CT scan of the chest and abdomen: (A) Chest CT illustrating a cavitary lesion in the right upper lobe with central consolidation, intralesional air, multiple areas of increased attenuation and regions of ground-glass opacity with heterogeneous distribution, findings consistent with active pulmonary tuberculosis. (B) Axial CT scan demonstrating marked mural thickening and luminal narrowing of small bowel loops, with adjacent mesenteric fat stranding suggestive of active inflammation. Multiple enlarged mesenteric lymph nodes with a hypodense center (characteristic of caseating necrosis) are visualized, consistent with tuberculous lymphadenitis. (C) Coronal abdominal CT scan illustrating multiple dilated small bowel loops with heterogeneous wall thickening, suggestive of inflammatory or infectious involvement. Notably, there is the presence of free intraperitoneal air adjacent to the left flank, indicative of a perforated viscus. Several mesenteric lymph nodes appear enlarged, consistent with reactive or granulomatous lymphadenopathy. These radiologic features are consistent with advanced abdominal tuberculosis complicated by intestinal perforation. CT, computed tomography.

Given the clinical context and the history of the patient, complementary analyses were requested. Viral serology yielded a positive result for hepatitis C, although with no detectable viral load. An abdominal computed tomography (CT) angiography ruled out mesenteric ischemia, and the patient was admitted to the Internal Medicine service at Hospital General Regional No 1, Unidad Morelos for further diagnostic workup. Upon admission, a physical examination revealed a rigid abdomen, the absence of bowel sounds and severe abdominal pain (10/10 on the visual analog scale). A non-contrast abdominal CT scan revealed dilated loops of small bowel, irregular mural thickening of the terminal ileum, moderate amounts of free peritoneal fluid, and mesenteric fat stranding, findings suggestive of secondary peritonitis due to intestinal perforation. A chest CT scan revealed a thick-walled cavitary lesion in the upper right lobe with central consolidation, internal air and bilateral areas of ground-glass opacity with heterogeneous distribution. These findings were compatible with active pulmonary tuberculosis (Fig. 1).

An exploratory laparotomy was performed, revealing the following intraoperative findings: A total of 100 ml of purulent free fluid, dilated small bowel loops, mural thickening, multiple interloop adhesions, intestinal granulomatosis, a 5-mm perforation located ~150 cm from the Treitz angle, indurated mesenteric lymph nodes, fibrotic rings in the ileum 50 cm from the ileocecal valve and cecal induration. The affected bowel segment was resected, followed by terminal ileostomy creation, mucous fistula formation and abdominal cavity lavage. The surgical specimens were submitted to the pathology laboratory of the hospital for comprehensive gross and histopathological analysis.

**Figure 2:**
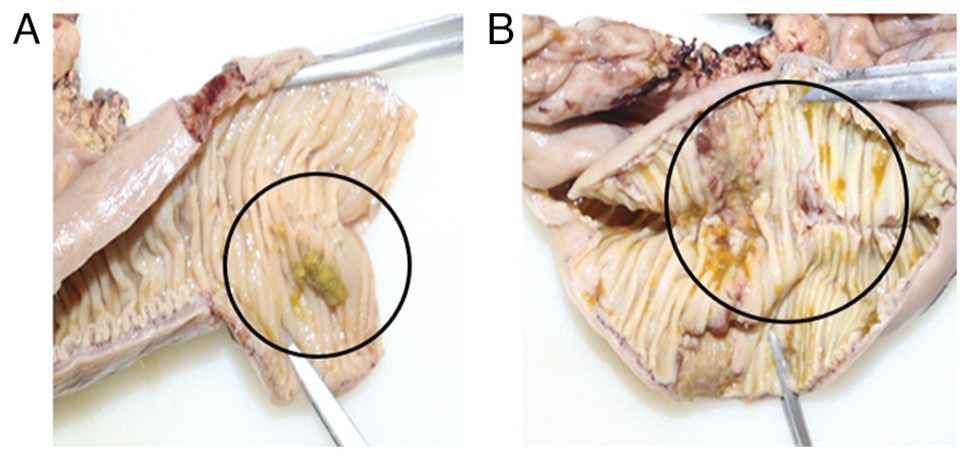
Macroscopic images of the resected intestinal segment. (A) A segment of the resected small intestine with focal mucosal ulceration and yellowish discoloration suggestive of caseating necrosis (black circle) is presented. The mucosal folds appear thickened, with evident loss of normal architecture in the affected area, indicative of localized granulomatous inflammation. (B) A more extensive segment is observed with diffuse mucosal thickening and multiple areas of yellowish exudate, consistent with widespread caseation (black circle). The transmural involvement and rigidity of the intestinal wall suggest chronic inflammation with possible fibrosis.

Histopathology reported ulcerated chronic granulomatous enteritis with caseous necrosis and intestinal perforation of tuberculous etiology. Surgical margins exhibited acute inflammatory changes with the formation of sinus tracts, indicating active infectious involvement rather than sterile perforation. The mesentery contained 14 lymph nodes with caseating granulomatous lymphadenitis, as well as multiple granulomas in the adjacent adipose tissue (Fig. 2). Ziehl-Neelsen staining revealed sparse acid-fast bacilli. Based on these findings, a diagnosis of intestinal tuberculosis with perforation was established, and treatment was initiated according to the DOTBAL regimen (rifampicin, 10 mg/kg/day; isoniazid, 5 mg/kg/day; pyrazinamide, 25 mg/kg/day; and ethambutol, 15 mg/kg/day), supplemented with vitamin B12 (1,000 µg/day). The patient was discharged with scheduled follow-up in the outpatient clinic.

## Discussion

The present case report describes the diagnostic approach in a middle-aged, immunocompetent male with risk factors for tuberculosis who presented with chronic, nonspecific abdominal symptoms that culminated in intestinal perforation secondary to undiagnosed abdominal tuberculosis.

Given the clinical and pathological findings, a comprehensive review of similar cases and diagnostic challenges is warranted. Reports in the literature have documented similar presentations of intestinal tuberculosis in immunocompetent adults, often involving chronic, vague abdominal symptoms that mimic other gastrointestinal pathologies. The review article by Debi *et al* (16) indicated that the ileocecal region is the most commonly affected site, with perforation occurring in <5% of cases, typically in advanced or undiagnosed disease. In the study by Ahmed *et al* (17), patients with intestinal tuberculosis presented with abdominal pain, altered bowel habits, fever and weight loss, features that overlap with malignancies or inflammatory bowel diseases, such as Crohn's disease. Moreover, the majority of cases required surgical exploration for definitive diagnosis, as preoperative imaging and laboratory tests often failed to confirm the etiology (17).

In the case series by Chan and Lee (18), several patients without HIV or other known causes of immunosuppression presented with perforation due to intestinal tuberculosis, with diagnosis achieved only postoperatively via histopathology. These findings align with the present case, where the lack of specific clinical indicators, the gradual onset of symptoms, and a non-specific response to initial symptomatic treatment delayed appropriate intervention. This underscores the necessity of considering abdominal tuberculosis in the differential diagnosis of chronic abdominal pain, even in the absence of clear immunosuppressive factors, particularly in regions with an intermediate or high prevalence of tuberculosis.

Additionally, the increasing incidence of atypical and extrapulmonary tuberculosis in younger populations without classic risk factors highlights the evolving epidemiological landscape of this disease (19). This reinforces the importance of a multidisciplinary diagnostic strategy and heightened clinical suspicion, particularly when non-specific gastrointestinal symptoms are accompanied by weight loss, systemic signs, or a suggestive family or epidemiological background.

Tuberculosis is an infectious disease known since antiquity; its causative agent was identified in the 19th century, a period marked by significant disease spread. Due to its predominant respiratory manifestations, tuberculosis was soon recognized as one of the first major global public health challenges (20). Although its incidence has declined in recent decades, tuberculosis remains one of the leading causes of mortality from infectious diseases. Currently, up to 15% of all cases are extrapulmonary, with abdominal tuberculosis being the most common form, prompting the WHO to prioritize its early identification (21).

Abdominal tuberculosis may result from the direct ingestion of the pathogen or hematogenous dissemination from a primary pulmonary focus, mediated by immune cells (22). Once in the abdominal cavity, *Mycobacterium tuberculosis* can affect the gastrointestinal tract, peritoneum, solid abdominal organs and mesenteric lymph nodes. Although it shares risk factors with pulmonary tuberculosis, it also presents distinct characteristics, being more commonly observed in women and in patients with chronic conditions that induce a pro-inflammatory state, even in the absence of overt immunosuppression (23).

In the case presented herein, the identified risk factors did not fully align with those typically described for abdominal tuberculosis. The absence of prolonged, specific symptoms and the abrupt clinical worsening hindered the initial diagnosis. This aligns with previous reports highlighting the difficulty of establishing an etiologic diagnosis in immunocompetent individuals, where confirmation is achieved only after a thorough clinical history is obtained (24,25). Similar cases have been described in patients of comparable age and immune status, without evident risk factors, who developed acute disease following nonspecific constitutional and gastrointestinal symptoms. In such instances, diagnosis was only reached after comprehensive imaging, serologic testing and histopathologic evaluation (26).

Beyond clinical features, the patient in the present study exhibited unconventional, yet relevant risk factors, such as high-risk behaviors and a history of poor hygiene (27,28). Additionally, prior epidemiological research has reported that in northern and northeastern regions of Mexico, where the patient was originally from, latent or oligosymptomatic tuberculosis is prevalent, particularly among young and middle-aged males, possibly explaining the primary infection in this case (29).

Although the patient described herein did not present with respiratory symptoms, imaging analyses revealed a pulmonary cavitation, consistent with latent tuberculosis. This finding may contribute to the development of extrapulmonary forms of the disease (30). Household exposure, particularly the history of a sister who succumbed due to pulmonary tuberculosis, supports the hypothesis of prior latent infection (31). Notably, a number of young patients discontinue prophylactic treatment or follow-up, even when a direct family history is present, particularly in contexts lacking support networks or with gaps in epidemiologic surveillance (32).

It is also worth noting that the onset of symptoms in this patient coincided with the COVID-19 pandemic in Mexico. In this context, it is reasonable to consider that the tuberculosis diagnosis may have been delayed due to prioritization of COVID-19 cases and the suspension of screening programs in high-risk populations (33). While the pandemic highlighted the importance of infectious diseases, it also negatively affected the care of other conditions, particularly respiratory illnesses (34). The strain placed on epidemiological surveillance systems led to under-diagnosis and clinical deterioration in patients with tuberculosis, which may have influenced the outcome in the case described herein (35).

The present case report is subject to several limitations. First, the diagnosis of intestinal tuberculosis was only confirmed post-operatively through histopathology, as microbiological cultures or PCR testing were not performed. Second, the clinical course of the patient was documented over a limited follow-up period, which restricts the evaluation of a long-term treatment response. Additionally, healthcare disruptions during the COVID-19 pandemic may have contributed to diagnostic delays. As with all single-case reports, generalizability to broader populations is limited.

Although detecting latent tuberculosis could aid in identifying non-specific clinical presentations and preventing extrapulmonary forms, its diagnosis remains challenging. The wide variability in the sensitivity of available tests, compounded by diverse immunological and inflammatory conditions in the Mexican population, limits the feasibility of mass screening strategies (36). Nevertheless, the complex interaction between *Mycobacterium tuberculosis* and its host promotes mutations that confer antimicrobial resistance (37). The rising prevalence of multidrug-resistant strains has renewed interest in the follow-up and treatment of latent tuberculosis infections (38). In this context, case reports, such as the present one underscore the need to strengthen adherence to therapeutic and prophylactic regimens among contacts of diagnosed patients, and to consider tuberculosis in the differential diagnosis of acute abdominal syndromes, even in young, immunocompetent men with no specific clinical history (39). The present case report serves as a valuable reminder of the varied presentations of tuberculosis and the need for vigilance even in non-immunocompromised individuals.

In conclusion, intestinal tuberculosis remains a diagnostic challenge, particularly in immunocompetent patients presenting with non-specific abdominal symptoms and no clear history of recent exposure. The case described in the present study highlights the importance of maintaining a high index of clinical suspicion in atypical abdominal presentations, particularly in endemic regions and among individuals with unconventional risk factors, such as poor hygienic conditions or a family history of tuberculosis. The surgical outcome resulting from an advanced form of abdominal tuberculosis underscores the need to strengthen early detection systems, particularly in settings where epidemiologic surveillance has been weakened, such as during the COVID-19 pandemic. The timely identification of latent tuberculosis and adherence to prophylactic treatment protocols could help prevent severe complications, including intestinal perforation. The present case report emphasizes the value of a multidisciplinary approach and the combined use of clinical, radiological and histopathological tools for the comprehensive diagnosis of this condition.

## Data Availability

The data generated in the present study may be requested from the corresponding author.

## References

[1] Chaudhary P, Kumar R, Ahirwar N, Nabi I, Gautam S, Munjewar C, Kumar A (2016). A retrospective cohort study of 756 cases of abdominal tuberculosis: Two decades single centre experience. Indian J Tuberc.

[2] Udgirkar S, Jain S, Pawar S, Chandnani S, Contractor Q, Rathi P (2019). Clinical profile, drug resistance pattern and treatment outcomes of abdominal tuberculosis patients in western india. Arq Gastroenterol.

[3] Ionescu S, Nicolescu AC, Madge OL, Marincas M, Radu M, Simion L (2021). Differential diagnosis of abdominal tuberculosis in the adult-literature review. Diagnostics (Basel).

[4] https://www.who.int/teams/global-tuberculosis-programme/tb-reports/global-tuberculosis-report-2022.

[5] Ordaz-Vázquez A, Torres-González P, Cruz-Hervert P, Ferreyra-Reyes L, Delgado-Sánchez G, García-García L, Kato-Maeda M, Ponce-De-León A, Sifuentes-Osornio J, Bobadilla-Del-Valle M (2021). Genetic diversity and primary drug resistance transmission in Mycobacterium tuberculosis in southern Mexico. Infect Genet Evol.

[6] https://www.cdc.gov/tb/publications/factsheets/statistics/tbtrends.html.

[7] Busatto C, Bierhals DV, Vianna JS, Silva PEAD, Possuelo LG, Ramis IB (2022). Epidemiology and control strategies for tuberculosis in countries with the largest prison populations. Rev Soc Bras Med Trop.

[8] Cheng MP, Abou Chakra CN, Yansouni CP, Cnossen S, Shrier I, Menzies D, Greenaway C (2017). Risk of active tuberculosis in patients with cancer: A systematic review and meta-analysis. Clin Infect Dis.

[9] Jha DK, Pathiyil MM, Sharma V (2023). Evidence-based approach to diagnosis and management of abdominal tuberculosis. Indian J Gastroenterol.

[10] Wenting J, Yuyan M, Qingfeng S, Yao Z, Yumeng Y, Yi S, Yingnan H, Qing M, Qingqing W, Mengran W (2022). Clinical features and diagnostic approaches for abdominal tuberculosis: Five-year experience from a non-tuberculosis-designated hospital in China. Rev Esp Enferm Dig.

[11] Das CJ, Rednam N, Vora Z, Aggarwal A, Chandrashekhara SH, Kundra V (2023). Abdominal visceral tuberculosis: A malignancy mimic. Abdom Radiol (NY).

[12] Dahale AS, Dalal A (2022). Evidence-based commentary: Ascitic adenosine deaminase in the diagnosis of peritoneal tuberculosis. J Gastrointest Infect.

[13] Brehm TT, Schmiedel S, Lohse AW (2022). Diagnosis of abdominal tuberculosis by mini-laparoscopy. Infection.

[14] Stein CM (2023). Genetic epidemiology of resistance to M. tuberculosis Infection: Importance of study design and recent findings. Genes Immun.

[15] Jena A, Mohindra R, Rana K, Neelam PB, Thakur DC, Singh H, Gupta P, Suri V, Sharma V (2023). Frequency, outcomes, and need for intervention in stricturing gastrointestinal tuberculosis: A systematic review and meta-analysis. BMC Gastroenterol.

[16] Debi U, Ravisankar V, Prasad KK, Sinha SK, Sharma AK (2014). Abdominal tuberculosis of the gastrointestinal tract: Revisited. World J Gastroenterol.

[17] Ahmed S, Hafez W, El Chayeb J, Al Jassem N, Massoud A, Nader S, Aboushady R (2023). Intestinal tuberculosis and inflammatory bowel disease; the usual challenging differential diagnoses: A case report. Radiol Case Rep.

[18] Kok-Hong Chan D, Lee KC (2015). Perforated intestinal tuberculosis in a non-AIDS immunocompromised patient. Am J Case Rep.

[19] Rolo M, González-Blanco B, Reyes CA, Rosillo N, López-Roa P (2023). Epidemiology and factors associated with Extra-pulmonary tuberculosis in a Low-prevalence area. J Clin Tuberc Other Mycobact Dis.

[20] Barbier M, Wirth T

[21] https://iris.who.int/handle/10665/373828.

[22] Cho JK, Choi YM, Lee SS, Park HK, Cha RR, Kim WS, Kim JJ, Lee JM, Kim HJ, Ha CY (2018). Clinical features and outcomes of abdominal tuberculosis in southeastern Korea: 12 years of experience. BMC Infect Dis.

[23] Al-Zanbagi AB, Shariff MK (2021). Gastrointestinal tuberculosis: A systematic review of epidemiology, presentation, diagnosis and treatment. Saudi J Gastroenterol.

[24] Niu T, He F, Yang J, Ma C, Xu J, Sun T, Zhang X, Chen S, Ru C (2023). The epidemiological characteristics and infection risk factors for extrapulmonary tuberculosis in patients hospitalized with pulmonary tuberculosis infection in China from 2017 to 2021. BMC Infect Dis.

[25] Mirijello A, Ritrovato N, D'Agruma A, de Matthaeis A, Pazienza L, Parente P, Cassano DP, Biancofiore A, Ambrosio A, Carosi I (2023). , *et al*: Abdominal lymphadenopathies: Lymphoma, brucellosis or tuberculosis? Multidisciplinary approach-case report and review of the literature. Medicina (Kaunas).

[26] Meregildo-Rodriguez ED, Tafur-Ramirez RC, Espino-Saavedra WG, Angulo-Prentice SF (2021). Abdominal tuberculosis misdiagnosed as acute surgical abdomen and carcinomatosis. F1000Res.

[27] Chien K, Seemangal J, Batt J, Vozoris NT (2018). Abdominal tuberculosis: A descriptive case series of the experience in a Canadian tuberculosis clinic. Int J Tuberc Lung Dis.

[28] Curry J, Abdelbary B, Garcia-Viveros M, Garcia JI, Yotebieng M, Rendon A, Torrelles JB, Restrepo BI (2022). South to north migration patterns of tuberculosis patients diagnosed in the Mexican border with Texas. J Immigr Minor Health.

[29] Flores-Aréchiga A, Zacarías-Hernández JL, Vázquez-Cortés CG, Tamez-Guerra RS, De la O-Cavazos M, Rivera-Morales LG, Llaca-Díaz JM, Castro-Garza J, Casillas-Vega N, Vázquez-Guillén JM, Rodríguez-Padilla C (2023). Molecular epidemiology and drug resistance of Mycobacterium tuberculosis in a tertiary care hospital in northeastern Mexico. J Infect Dev Ctries.

[30] Schwartz NG, Price SF, Pratt RH, Langer AJ (2020). Tuberculosis-United States, 2019. MMWR Morb Mortal Wkly Rep.

[31] Behr MA, Edelstein PH, Ramakrishnan L (2018). Revisiting the timetable of tuberculosis. BMJ.

[32] Medrano BA, Lee M, Gemeinhardt G, Rodríguez-Herrera JE, García-Viveros M, Restrepo BI (2023). Tuberculosis presentation and outcomes in older Hispanic adults from Tamaulipas, Mexico. Medicine (Baltimore).

[33] Muñiz-Salazar R, Le T, Cuevas-Mota J, González-Fagoaga JE, Zapata-Garibay R, Ruiz-Tamayo PS, Robles-Flores J, Garfein RS (2022). Impact of COVID-19 on tuberculosis detection and treatment in Baja California, México. Front Public Health.

[34] Bi K, Cao D, Ding C, Lu S, Lu H, Zhang G, Zhang W, Li L, Xu K, Li L, Zhang Y (2022). The past, present and future of tuberculosis treatment. Zhejiang Da Xue Xue Bao Yi Xue Ban.

[35] Dheda K, Perumal T, Moultrie H, Perumal R, Esmail A, Scott AJ, Udwadia Z, Chang KC, Peter J, Pooran A (2022). The intersecting pandemics of tuberculosis and COVID-19: population-level and patient-level impact, clinical presentation, and corrective interventions. Lancet Respir Med.

[36] Carranza C, Pedraza-Sanchez S, de Oyarzabal-Mendez E, Torres M (2020). Diagnosis for latent tuberculosis infection: New alternatives. Front Immunol.

[37] Liebenberg D, Gordhan BG, Kana BD (2022). Drug resistant tuberculosis: Implications for transmission, diagnosis, and disease management. Front Cell Infect Microbiol.

[38] Esaulova E, Das S, Singh DK, Choreño-Parra JA, Swain A, Arthur L, Rangel-Moreno J, Ahmed M, Singh B, Gupta A (2021). The immune landscape in tuberculosis reveals populations linked to disease and latency. Cell Host Microbe.

[39] Rahman SMM, Rahman A, Nasrin R, Ather MF, Ferdous SS, Ahmed S, Uddin MKM, Khatun R, Sarker MS, Mahmud AM (2022). Molecular epidemiology and genetic diversity of multidrug-resistant mycobacterium tuberculosis Isolates in Bangladesh. Microbiol Spectr.

